# Enhancement of Microencapsulation of Rapeseed Oil Bioactive Compounds in Alginate Through Sonication

**DOI:** 10.3390/foods14101692

**Published:** 2025-05-10

**Authors:** Cristina-Emanuela Enascuta, Elena-Emilia Sirbu, Diana Pasarin, Andra Ionela Ghizdareanu, Raluca Senin, Ioana Silvia Hosu, Ana-Mihaela Gavrilă, Bianca-Ana-Maria Burdusel, Vasile Lavric

**Affiliations:** 1 National Institute for Research & Development in Chemistry and Petrochemistry ICECHIM, 202 Splaiul Independenței Str., 060021 Bucharest, Romania; cristina.enascuta@gmail.com (C.-E.E.); diana.pasarin@gmail.com (D.P.); ghizdareanuandra@gmail.com (A.I.G.); raluca.senin@icechim.ro (R.S.); ioana.hosu@icechim.ro (I.S.H.); anamihaela.florea@gmail.com (A.-M.G.); 2 Chemistry Department, Petroleum-Gas University of Ploiești, Bucharest Blvd. 39, 100680 Ploiești, Romania; bya.aldea@yahoo.com; 3 Faculty of Chemical Engineering and Biotechnologies, National University of Science and Technology Politehnica Bucharest, 011061 Bucharest, Romania

**Keywords:** microencapsulation process, alginate microcapsules, rapeseed oil, bioactive compounds, ultrasound-assisted field, experiment design

## Abstract

The microencapsulation of bioactive compounds from rapeseed oil using sodium alginate, in the presence and absence of an ultrasonic (US) field, is reported. A Box–Behnken experimental design is used to investigate the influence of process parameters on the microencapsulation yield; then, the response surface methodology is applied, to find their values ensuring its optimum yield. The operating parameters investigated are the ratio of sodium alginate to rapeseed oil, the microencapsulation time and the concentration of the calcium chloride solution. The US bath was used at its nominal power, and the microencapsulation temperature was kept at 20 °C, with a thermostat, for all experiments. A detailed study on the comparison of the two microencapsulation techniques (in the presence and absence of the US field) was carried out. Good results were obtained in the presence of the US field for optimal conditions, when the microencapsulation yield was 90.25 ± 0.02%, higher than the microencapsulation process performed in the absence of the US field, 87.11 ± 0.02%. The results also showed that the use of the US field (optimal conditions) led to an increase in encapsulation efficiency, total phenolic content and antioxidant capacity (76.56 ± 0.02%, 324.85 ± 0.01 mg GAE/g and 57.05 ± 0.12 mg/mL). The physicochemical description of microcapsules was performed using modern characterization methods. These results indicate that by increasing the microencapsulation yield of bioactive compounds through sonication, the process aims to achieve a uniform size distribution of microcapsules.

## 1. Introduction

In recent years, consumers’ growing demand for high-quality, minimally processed products highlighted the use of plant-based alternatives. To meet these expectations, one option is the utilization of encapsulated bioactive compounds. By incorporating these compounds into various matrices, it is possible to obtain functionalized products with enhanced benefits, nutraceuticals or dietary supplements [1,2]. Rapeseed oil is considered one of the healthiest vegetable oils, due to its content of bioactive compounds that offer many health benefits. The latest reports show that rapeseed has four major production areas: China, India, Canada and the European Union. At the European level, the demand for the cultivation of this plant remains strong enough to support a continuous progression in the rapeseed oil production industry [3]. This oil is one of the most consumed edible oils in the world, being a rich source of mono (α-linoleic acid) and polyunsaturated fatty acids (omega-3/omega-6) that the body cannot produce, vitamin E, phytosterols, antioxidant compounds and carotenoids. Thanks to these bioactive compounds, rapeseed oil has beneficial effects on cardiovascular health, has anti-inflammatory and antioxidant properties, supports brain function and helps maintain a healthy immune system. Also, due to the optimal ratio of polyunsaturated fatty acids, it is considered a nutritionally balanced oil [4]. Microencapsulation is a technological process of forming a functional barrier between the core material and the environment, the wall shell. The compounds captured in the core material are thus protected from factors that can lead to degradation or loss of efficacy, such as oxygen, humidity, light or high temperatures [5]. By improving stability, bioavailability and controlled release of bioactive compounds such as omega-3 fatty acids, antioxidants and vitamins, microencapsulated rapeseed oil can be effectively used in dietary supplements, functional foods and beverages. This technique not only protects sensitive nutrients but also increases their potential health benefits, making it an attractive option for the nutraceutical industry [6]. Because the rapeseed oil is physically trapped in the capsule matrix, a semi-solid/solid consistency is achieved, preserving its valuable nutritional value [7]. Capsule morphologies can vary greatly depending on the core and shell materials, as well as the encapsulation technique used [8]. For the efficient delivery of functional compounds, carrier systems should exhibit good properties such as absorption, prolonged delivery time, no clinical side effects and high biocompatibility. Various materials such as chitosan, gelatin, soy protein, pea, whey or cyclodextrin are used as carrier systems. Among the biodegradable polymers, alginate can be used successfully because the capsules can be easily prepared under mild conditions without solvents. Also, sodium alginate is quite often used as a coating material because it exhibits high strength and has considerable effects on the mechanical stability of capsules [6]. Alginate microcapsules are formed by a chemical gelation reaction based on the replacement of sodium ions with calcium ions, resulting in a three-dimensional matrix. This process allows the creation of microcapsules with high oil content that can be used in the food industry to improve food quality and generate functional food products [9]. Applications that involve the use of an ultrasonic field for various processing methods in the food industry (extraction, encapsulation, crystallization, emulsification, etc.) are experiencing continuous growth. US technology is effective in creating encapsulated materials with specific properties, thanks to the mechanical and cavitation effects it produces [10]. The wide range of active ultrasound frequencies enables precise modulation of cavitation dynamics, allowing manipulation of material such as particle size, surface texture or internal structure [11]. In this sense, the US-assisted technique is considered a good alternative to conventional phytochemical encapsulation techniques, being used as an independent method or in combination with other methodologies [1]. There are few studies in the literature on the microencapsulation of rapeseed oil rich in bioactive compounds, but other microencapsulation techniques are used. A recent study evaluated the nanoemulsion of rapeseed oil by sonication to obtain stable nanoemulsion gels based on proteins and starch [12]. The effect of changes in the emulsion compositions on the drying process on the efficiency of oil microencapsulation and on selected physical properties of the powder were analyzed [13].

In this research, alginate was used as the entrapment material and rapeseed oil, rich in bioactive compounds, was the entrapped liquid. The emulsion of rapeseed oil into sodium alginate solution was dripped, using a high-pressure syringe pump, into calcium chloride solution in the presence and absence of an US field. A Box–Behnken experimental design was used to investigate the influence of process parameters on the microcapsules’ properties. After that, the surface response methodology was applied to find the appropriate operating conditions ensuring the optimum encapsulation yield. The operating parameters investigated were the ratio of sodium alginate to rapeseed oil, the microencapsulation time (dripping and hardening) and the concentration of the calcium chloride solution, the latter being subjected or not to a US field. The physicochemical properties of the microcapsules were characterized and analyzed using Fourier transform infrared spectroscopy (FTIR), scanning electron microscopy (SEM), differential scanning calorimetry (DSC) and digital microscope. In addition, polyphenol content, antioxidant activity, encapsulation efficiency and fatty acid methyl esters (FAMEs) distribution were quantified.

The innovative aspect of our study focuses on exploring the effects of an US field on the microencapsulation process, compared to the classical one, in terms of yield, encapsulation efficiency, total phenolic content, antioxidant capacity, structure and uniform size distribution.

## 2. Materials and Methods

**Figure 1:**
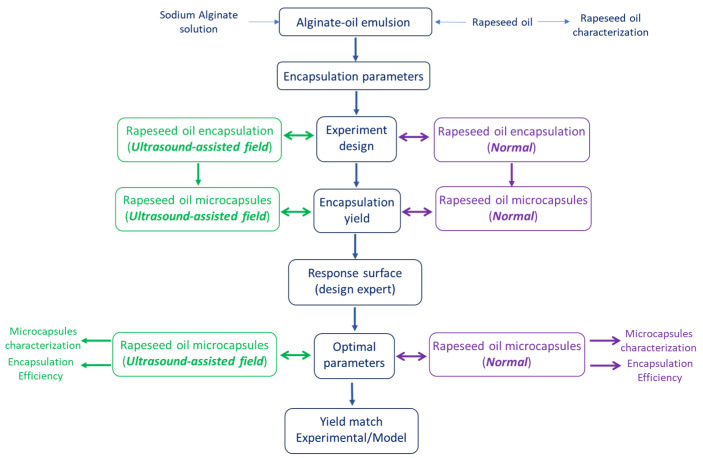
Block diagram of the sequence of procedures.

The block diagram (Figure 1) shows the steps followed to perform the experiments, characterize the products and find the optimal values for the operating parameters corresponding to the highest encapsulation yield.

### 2.1. Materials and Reagents

Sodium alginate, calcium chloride (98%), n-hexane (96), Folin–Ciocâlteu reagent and gallic acid were purchased from Sigma-Aldrich (Taufkirchen, Germany). Potassium chloride (99.5%), sodium carbonate (99.5%), ethanol (96%) and chloroform (99%) were purchase from SC Chimreactiv SRL (Bucharest, Romania). The rapeseed oil was obtained by inhouse US-assisted extraction from the rapeseed seeds in a reaction setup already presented [14]. It contained a total amount of mono- and polyunsaturated fatty acid of about 86% (*w*/*w*). Its density was 0.95 g/mL. The total phenolic content in the crude oil product, without encapsulation, was 287.88 mg GAE/g and the antioxidant activity (IC50) of the same oil was 51.74 mg/mL.

### 2.2. Working Protocol

The sodium alginate solution with a concentration of 2% (*w*/*w*) was obtained by dissolving sodium alginate in distilled water under magnetic stirring (500 rpm) at room temperature (20 °C) for approximately 30 min. The solution was left to stand overnight to remove air bubbles.

According to the data generated by the experimental program, twenty milliliters of alginate/oil emulsion were uniformly dropped into calcium chloride solutions of different concentrations in the presence of a US field or without it (magnetic stirring 500 rpm) using a high-pressure syringe pump (NE-1010,KFT, Rome, Italy) with a 0.7 mm nozzle and a 5 cm distance from the dispense tip to the surface of the calcium chloride solution. The formed microcapsules were allowed to harden for 30 min in the sodium chloride bath, irrespective of the dripping time; then, they were filtered and washed twice with distilled water, then dried on filter paper to remove traces of water and oil from the surface of the wet capsules.

US-assisted microencapsulation was performed using a high-power ultrasonic bath with 160 W nominal power and 35 kHz frequency (Sonorex DigiPlus DL 255 H, Bandelin Electronic GmbH & Co. KG, Berlin, Germany) coupled with a compact recirculating cooler (IKA RC 2 lite, IKA Works GmbH & Co, Staufen, Germany). In all experiments (labelled US), the intensity of the sonication corresponded to the nominal power of the bath. The microencapsulation temperature was maintained at 20 °C, while sonication lasted for the dripping and hardening time. The microencapsulation process was also carried out in the absence of a US field, the experiments being labelled Control.

The choice of concentrations of sodium alginate and calcium chloride solutions was based on relevant published studies and on the acquired experience of the authors of this article, in which the amount of encapsulated oil and the release patterns of the encapsulated compounds were determined based on experimental data on the encapsulation efficiency [15,16]. To quantify the replicability of the results, all experiments were performed in duplicate, characterized by standard deviations lower than 3%.

### 2.3. Response Surface Methodology

Response surface methodology (RSM), as implemented in Design-Expert (DE) software, was used to optimize the process parameters of the encapsulation of rapeseed oil rich in bioactive compounds in the presence or absence of a US field. RSM uses empirical second-order polynomials (Equation (1)) with interactions to model the connections between the operating parameters and the process performance, encapsulation yield and efficiency, in this case.

(1) $$y=a+\sum_{i=1}^{n}b_{i}\cdot x_{i}+\sum_{i=1}^{n-1}\sum_{j=i}^{n}c_{i,j}\cdot x_{i}\cdot x_{j}+\sum_{i=1}^{n}d_{i}\cdot x_{i}^{2}$$

Here, *n* is the number of the operating parameters, *a*, *b*, *c* and *d* are the coefficients of the second order polynomial (unknowns), *x* stands for the operating parameters, while *y* is the targeted performance.

The Box–Behnken (BB) experimental design, providing the necessary number of experimental points, *n*, gave the values of the operating parameters for which the encapsulation yields/efficiencies were found. According to the BB technique, the minimum number of factors is three (min, middle, max), with four replicas in the center of the experimental field.

The coefficients of the second-order polynomials were obtained by regression analysis over the experimental yields/efficiencies, found in the experimental points provided by the Box–Behnken technique. Response surface diagrams were made by DE and the relationships between factors were analyzed.

### 2.4. Encapsulation Yield and Efficiency

The encapsulation yield was calculated with Equation (2):

(2) $$Y=\frac{Mc}{Mi}\times 100\;\;\;\;\;$$

where *Y* is the encapsulation yield, %, *Mc* is the mass of the obtained rapeseed oil microcapsules, g, and *Mi* is the initial mass of the alginate/oil emulsion dripped in the calcium chloride solution, g.

The encapsulation efficiency (EE) of microcapsules loaded with rapeseed oil rich in bioactive compounds was measured following the method of Gulzar et al. with minor modifications [17]. The amount of oil on the surface of the capsules and the total oil in the capsules were recovered and quantified. The surface oil was recovered by mixing 1 g of capsules with 10 mL of hexane. The mixture was homogenized (800 rpm) for 10 min at room temperature, then filtered through a filter paper. The oil extraction was repeated three times. The collected hexane fractions were evaporated on a rotary vacuum evaporator (Hei-VAP Advantage, Heidolph Instruments, Schwabach, Germany). The amount of oil on the surface of the capsules was calculated as the difference between the initial and final amount in the flask containing the combined layers of hexane/oil mixture. Total oil was measured by dissolving 1 g of microcapsules in 3 mL of 1% (*w*/*v*) potassium chloride solution, 5 mL methanol and 10 mL chloroform. The mixtures were homogenized for 10 min at 800 rpm, then transferred to a separatory funnel. The chloroform layer was collected and removed by evaporation on a rotary evaporator. The encapsulation efficiency was calculated with Equation (3):

(3) $$EE=\frac{To-So}{To}\times 100\;$$

where *EE* is the encapsulation efficiency, %, *To* is the total oil mass, g and *So* is the surface oil mass, g.

### 2.5. Fatty Acids Methyl Esters

Fatty acid methyl esters (FAMEs) of the extracted oil were prepared via transesterification for fatty acid compositional determination according to Murthy et al. [18].

The distribution of fatty acid methyl esters (FAMEs) was found using a GC–MS system (CLARUS 500, Perkin Elmer, Shelton, WA, USA), according to the method of Enascuta et al. using a capillary column Elite-5 MS, (60 m length, 0.32 mm internal diameter, 0.25 mm film thickness) and helium as carrier gas at 1 mL/min [19]. The oven temperature was initially set at 50 °C, then increased gradually to 250 °C, with 10 °C per minute and 5 min holding time. The GC injector and MS ion source temperatures were 250 °C. The transfer line temperature was 280 °C. The MS detector was operated in EI mode at 70 eV, with an m/z scanning range of 50–450. The identification of the FAMEs present in the rapeseed oil subjected to encapsulation was carried out using the NIST MS database. The area under the peaks was used for quantitative determination of the fatty acid percentages.

The determination of the fatty acids characteristics was performed according to the adapted European standard SR EN 14103:2003—fatty acid methyl esters (FAME). TurboMass 6.1 software was used for online driving of the instrument and for data processing.

### 2.6. Microcapsules Structure Characterization

The morphology of microcapsules was investigated using Scanning Electron Microscopy (SEM). SEM images for the reference and microcapsules loaded with rapeseed oil samples were recorded on a Hitachi TM4000Plus II benchtop (Tokyo, Japan) at an accelerating voltage of 15 kV with a cooling stage. The equipment was also supplied with an image signal high-sensitivity backscattered electron detector (BSE), a vacuum mode conductor, TM4000 application software with Windows 10 Professional license, capable of magnifications up to ×1.00 k. Before SEM analysis, the microcapsules were placed on a carbon strip and the surface morphology as well as the inner morphology in their wet form were further analyzed. In this context, the samples were uniformly coated with gold by cold sputtering using a Sputter Coater Q150R ES Plus (Quorum; Cambridge, UK) to create an electrically conductive thin film (~5 nm film thickness); this step was necessary to inhibit the “charge up”, thus reducing thermal damage and increasing the emission of secondary electrons. The wet microcapsules were perforated with a special needle for analyzing the inner morphology.

### 2.7. Microcapsule Morphology Analysis

To determine the dimensions of the microcapsules obtained under optimal conditions (in the presence and absence of the US field) and to observe their shape, an Optika B-150D-BRP digital microscope (Optika Microscopes, Ponteranica, Italy) equipped with image analysis software was used. Empty microcapsules obtained under the same conditions were also studied. The brightness and the magnification (25×) were adjusted to obtain the best focus for the acquired optical images. In addition, the average diameter of the microcapsule was measured from two different spots/perspectives. Also, the sphericity factor (SF) was used for the roundness of the microcapsules, according to [20]. The SF was calculated with Equation (4):

(4) $$SF=\frac{Dmax-Dper}{Dmax+Dper}$$

where *Dmax* is the maximum diameter passing through the bead (mm) and *Dper* is the perpendicular diameter to *Dmax* passing through the bead (mm).

Microcapsules with a value <0.05 were considered spherical, while higher values indicate a higher degree of shape distortion. All samples were tested in duplicate to determine the mean error. The shape and size measurements of the samples were determined on 15 microcapsules per sample.

### 2.8. Thermogravimetric Analysis

Thermogravimetric analysis (TGA) of samples was studied using a thermogravimetric/derivative equipment TGA/DTG (TGA 2-Star System, Mettler Toledo, Zurich, Switzerland). For each analysis, approximately 7 mg of sample was placed onto an alumina crucible of 70 mL, and heated at a constant rate of 10 °C/min from 25 to 800 °C, under nitrogen with a mass flow of 20 mL/min.

### 2.9. ATR-FTIR Spectral Measurements

The molecular structures and changes in the chemical structure of the sodium alginate, rapeseed oil and microcapsules loaded with rapeseed oil were compared and analyzed using an FTIR spectrometer (Bruker, Model Tensor 27) equipped with a 45 °C ZnSe ATR accessory (Bruker, Siegsdorf, Germany). The samples were added directly onto the ZnSe ATR crystal for spectral measurements.

Before proceeding to scan the sample, the background spectrum of the bare ATR crystal was collected for normalization of the spectrum intensity.

After each measurement, the ATR crystal was carefully cleaned with ethanol to remove the presence of oil residues. ATR-FTIR spectra were recorded using OPUS Ver. 7.5.18 software in the wavelength range 650–4000 cm^−1^ using 32 scans per sample at a resolution of 4 cm^−1^, with a mirror speed of 0.32 cm/s.

### 2.10. Total Phenol Content and Antioxidant Activity

The total phenol content was determined using the Folin-Ciocâlteu spectrophotometric method (Ultra 3600 Rigol), according to the procedure described previously by Kupina et al. [21]. Absorbances were measured at 765 nm, in triplicate. The results were expressed as milligrams of gallic acid equivalents per gram of microcapsules (mg GAE/g), based on the gallic acid calibration curve within the concentration range of 40–200 mg/mL. The total phenolic content (TP) was determined as follows (Equation (5)):

(5) $$TP=\frac{m}{A-b}\;\;\;\;\;$$

where *TP* is total phenolic content, mg GAE/g; *A* is the measured absorbance; *b* is the *y*-axis intercept of the calibration curve (*b* = 0.0538); *m* is the slope of the calibration curve (*m* = 0.0066, mg GAE/g).

The antioxidant activity of the rapeseed oil was measured using the 2, 2-diphenyl-1-picrylhydrazyl (DPPH) spectrophotometric method as described by Noreen et al. [22]. The decrease in absorbance of each sample was measured on the same spectrophotometer at 517 nm, in triplicate. Percentage DPPH inhibition was calculated using the formula (6):

(6) $$DPPH\;inhibition=\frac{Acontrol-Asample}{Acontrol}\times 100\;\;\;\;$$

where *DPPH inhibition*, %, *Acontrol* and *Asample* are the absorbance of the control and sample, respectively. The results were reported as IC50 values. A lower IC50 value represents a stronger DPPH scavenging capacity.

## 3. Results and Discussion

### 3.1. Operating Parameters Influence upon the Encapsulation Yield

To decide the operating parameters limits, which could contain the optimum encapsulation efficiency, we started from the idea that by keeping the flow rate constant (time of dripping), the droplets generated by the syringe dispense tip will have, regardless of the ratio, the same diameter. This means that, by increasing the ratio, the amount of oil in each capsule will be smaller (a disadvantage), but the capsule will form faster, thus the risk of losing oil will be lower (an advantage). Normally, the stability of the capsules should increase as the ratio increases, and the oil, in an increasingly smaller amount, for spherical capsules of the same diameter, will diffuse more slowly to the surface. Also important is the interaction between sodium alginate and CaCl_2_ which depends upon the abundance of Ca^2+^ ions in the solution. Considering that the formation of calcium alginate beads is an ion exchange reaction, the process should be quite fast even at lower concentrations of CaCl_2_ in the water.

The other important parameter of our process is the suspension flow rate through the syringe dispense tip, and hence, the drop formation time. The higher the flow rate, the smaller the droplet diameter, which could have small random variations, starting from a certain value. Due to their small diameters, oil can diffuse more easily to the surface. On the other hand, smaller flow rates imply higher droplet diameters, with possible implications for the release time.

According to the previous reasoning and to some experimental tests, the working values of the independent variables are the mass ratio of sodium alginate to rapeseed oil (5:1; 7.5:1; 10:1; *w*/*w*), the concentration of calcium chloride solution (1.5; 2.25; 3; *w*/*v*) and the dripping time (15; 22.5; 30; min). The US field intensity corresponds to the bath nominal power. The microencapsulation temperature was kept at 20 °C.

The impact of the sodium alginate/rapeseed oil ratio on the encapsulation yield (see Equation (1)) and the dripping time are presented in Table 1, in which the first three columns are the real levels of the corresponding coded ones given by the BB design. The results indicated that the encapsulation yield depends on these parameters.

For example, for the microencapsulation of rapeseed oil in a US field, the encapsulation yield reached 89.35 ± 0.03% at a sodium alginate/rapeseed oil ratio of 7.5, a calcium chloride concentration of 3% and a dripping time of 30 min. Also, the mass of the obtained microcapsules increases from 15.21 ± 0.02 g to 17.87 ± 0.04 g with the increase in the sodium alginate/rapeseed oil ratio from 5 to 7.5, the increase in calcium chloride concentration from 1.5 to 3% and the increase in the dripping time from 22.5 min to 30 min.

For the microencapsulation of rapeseed oil in the absence of a US field (Control), the encapsulation yield reached 86.40 ± 0.03% at a sodium alginate/rapeseed oil ratio of 7.5, calcium chloride concentration of 2.25% and dripping time of 22.5 min. Also, the mass of the obtained microcapsules increased from 15.16 ± 0.02 g to 17.28 ± 0.04 g with the decrease in the sodium alginate/rapeseed oil ratio from 10 to 7.5 and the increase in calcium chloride concentration from 1.5 to 2.25% for the same dripping time of 22.5 min. For longer dripping times (30 min), the experimental yields were smaller. Higher dripping times mean that the probability that the small drops of oil suspended in the alginate solution will coalesce increases, thus degrading the homogeneity of the suspension. During drop formation, larger drops will form inhomogeneous microcapsules, with possible increased coating defects, susceptible of losing, partly, some encapsulated oil. These results are supported by Soliman et al., who found that the efficiency of essential oil encapsulation is optimal at a microencapsulation time of around 20 min [16]. Prolonging this time, between 25–30 min, they noticed, as well, a decrease in efficiency of encapsulation.

### 3.2. Analysis Using RSM

RSM involves a regression analysis upon experimental data, to obtain the coefficients of the polynomials considered as empirical models, Equation (1). The yield of microencapsulation represents the dependent variable (Y), while the sodium alginate/rapeseed oil ratio (x_1_), the concentration of the calcium chloride solution (x_2_) and the dripping time (x_3_) are the independent variables. The resulting equations are as given by the MATLAB (R2024b-academic license) function *regstats*, which performs a polynomial regression of the responses in *Y* on the predictors in X, with the option to provide several diagnostic statistics. Amongst these, the *p*-values emphasize that all polynomial coefficients are statistically significant, *p* < 0.05.

(7) $$Y_{US}=-16.3+15.6\cdot x_{1}+19.8\cdot x_{2}+1.32\cdot x_{3}-0.27\cdot x_{1}\cdot x_{2}+0.038\cdot x_{1}\cdot x_{3}+0.013\cdot x_{2}\cdot x_{3}-0.99\cdot x_{1}^{2}-3.51\cdot x_{2}^{2}-0.029\cdot x_{3}^{2}$$

**Figure 2:**
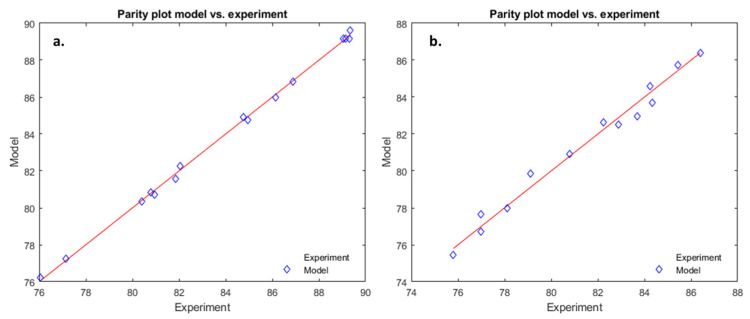
The parity plots for the microencapsulation process in the presence of the ultrasound-assisted field (US) (**a**) and the absence of the ultrasound-assisted field (Control) (**b**).

The coefficient of determination is 0.996 (see Figure 2a).

(8) $$Y_{C}=-12.9+9.04\cdot x_{1}+18.9\cdot x_{2}+3.11\cdot x_{3}-0.51\cdot x_{1}\cdot x_{2}+0.015\cdot x_{1}\cdot x_{3}-0.016\cdot x_{2}\cdot x_{3}-0.51\cdot x_{1}^{2}-2.84\cdot x_{2}^{2}-0.062\cdot x_{3}^{2}$$

The coefficient of determination is 0.962 (see Figure 2b).

In both cases, the operating parameters have a positive influence on the yield (positive coefficients for the linear terms), though they are higher for x_1_ and x_2_, but lower for x_3_. Regarding the interaction coefficients, in both cases, x_1_ and x_2_ have a rather small antagonistic effect, while x_1_ and x_3_ have an even smaller synergistic effect. In the case of microencapsulation subjected to the US field, x_2_ and x_3_ have an even smaller synergistic effect, while in the case of the Control process, they have an antagonistic effect, of the same order of magnitude. In both cases, the squared operating parameters all have an antagonistic effect upon the yield, with the same small order of magnitude.

This was an expected result since the process of microencapsulation is the same, except that the CaCl_2_ solution is subjected, or not, to the US field. The increase in the encapsulation yield could be attributed to the hardening process, faster in the US field, due to the changed hydrodynamic of both phases, the alternative movements generated by the US field decreasing the boundary layer surrounding the drops.

These two polynomials were, then, used to find the operating conditions for which the optimum yield is obtained, in both conditions. The results are presented in Table 2. As expected, the optimum values for the operating parameters of both cases are close to each other, a little bit smaller for the normal case. Quite interestingly, the dripping time is longer for the US experiment, suggesting that a greater yield corresponds to smaller drops hardening faster.

To verify the predictions of the model, two experiments (US and Control) were performed, using the optimal operating parameters, for which the corresponding experimental microencapsulation efficiencies (see Table 2) were 90.25 ± 0.02% in the presence of the US field (US), and 87.11 ± 0.02% in the absence of the ultrasound field (Control), respectively, both results being close to the predicted values. This constitutes a validation of the polynomial model.

### 3.3. Fatty Acid Composition

The distribution of fatty acids in microencapsulated rapeseed oil is presented in Table 3. The carbon chain length of each fatty acid ranges from 16 to 22. Due to the high proportion of unsaturated fatty acids, microencapsulated rapeseed oil may be preferred over other vegetable oils.

The present study showed that the content of monounsaturated fatty acids (MUFAs) in the encapsulated oil was very high (66.76 ± 0.1%), which is consistent with the results obtained by Tanska et al. [23]. Oleic acid is the most abundant MUFA present in rapeseed oil (61.76 ± 0.09%). In contrast, the amount of polyunsaturated fatty acids (PUFAs) particularly linoleic acid and linolenic acid was 24.72 ± 0.05%, while saturated fatty acids (SFAs) accounted for 8.52 ± 0.01%.

As reported by Guo et al. and Chew et al., the fatty acids in microencapsulated rapeseed oil are present in the following order: MUFA > PUFA > SFA [24,25].

### 3.4. ATR-FTIR Spectral Measurements

**Figure 3:**
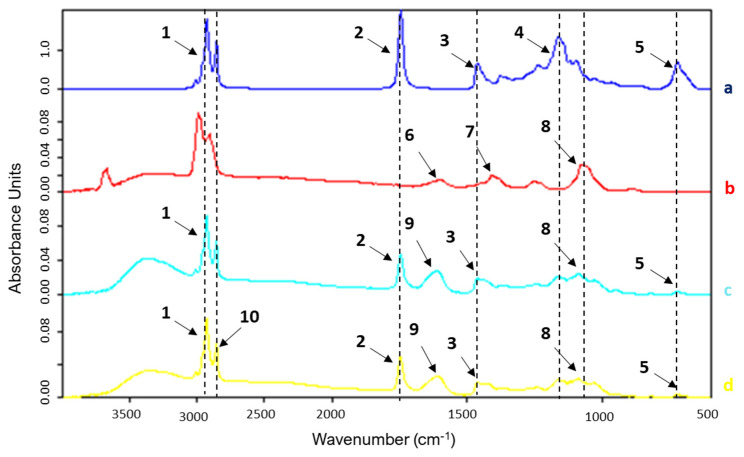
FTIR spectra of rapeseed oil (**a**), sodium alginate (**b**) and microcapsules obtained in the absence of the US field (Control) (**c**) and in the presence of the US field (US) (**d**).

Figure 3 and Table 4 show the FTIR spectra of sodium alginate (a), rapeseed oil (b) and microcapsules obtained in the absence of the US field (Control) (c) and in the presence of the US field (US) (d).

The FTIR spectra of sodium alginate exhibit bands with different intensities at 2923 cm^−1^ (1) which can be attributed to the CH_2_^−^ stretching vibration on saturated carbon atoms and at 2853 cm^−1^ (10), corresponding to the aliphatic (–CH–) group representing the degree of unsaturation of the rapeseed oil [5,17].

The absorption bands at 1628 cm^−1^ (6) (7) represent the asymmetric and symmetric carboxylate-induced (–COO–) stretching vibrations, while the bands at and 1393 cm^−1^ and 1066 cm^−1^ (8) could be attributed to the C–OH stretching vibration (Figure 3b). As Gulzar et al. also observed, the large absorption bands in the range 3600–3000 cm^−1^ from sodium alginate are due to the stretching vibrational band of the –OH groups in alginic acid [17].

Figure 3b shows the FTIR spectrum of rapeseed oil. Characteristic peaks for long-chain linear aliphatic compounds were observed between 2987 and 2853 cm^−1^ assigned to stretching vibrations in –CH_3_ or –CH_2_ groups, followed by two bands at 1461 cm^−1^ (3) and 742 cm^−1^ (5) resulting from the bending vibrations of –CH_2_ and the in-plane C–H oscillation vibration of the lipid carbon skeleton [26,27]. The strong band at 1744 cm^−1^ (2) corresponds to the stretching vibration of the fatty acid carbonyl C=O ester from triglyceride molecules of rapeseed oil; meanwhile, the band at 1160 cm^−1^ (4) could be assigned to the symmetric stretching vibration of the C–O–C bond in the fatty acid ester [24]. The FTIR spectra of the Control- and US-obtained microcapsules (Figure 3c,d) showed the characteristic absorption bands of sodium alginate (C–OH stretching vibration around 3355 cm^−1^ and 1068 cm^−1^ and 1635 cm^−1^ (9) assigned to carboxylate group) and rapeseed oil (1744 cm^−1^ and 1160 cm^−1^ due to C=O and C–O–C bonds from triglycerides) which confirms that the oil was successfully entrapped into the alginate microcapsules. The spectra of Control- and US-obtained microcapsules were similar, suggesting that the US field did not change the composition of the encapsulated oil. This suggests that, despite its presence, cavitation did not influence the encapsulation process, due to the coating barrier.

### 3.5. Thermogravimetric Analysis

**Figure 4:**
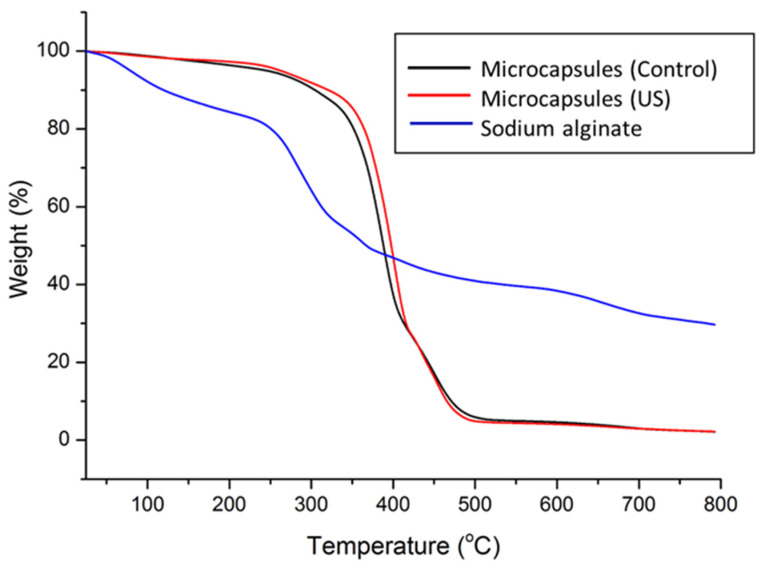
TGA thermograms for sodium alginate, microcapsules with rapeseed oil subjected to the US field and in the absence of the US field (Control).

Thermogravimetric analysis was carried out to investigate the influence of preparation condition on the thermal properties of sodium alginate microcapsules and microcapsules with rapeseed oil (in the presence of the US field (US) and the absence of the US field (Control)). As can be seen in Figure 4, all the samples had a similar pattern with three degradation stages. The first thermal step is observed for all samples in the temperature range of 25–150 °C and represents the loss of physically weak or chemically strong bound water [28]. The weight loss was 12.27% for sodium alginate, 1.85% for microcapsules with rapeseed oil in presence of the ultrasound-assisted field (US) and 2.34% for microcapsules with rapeseed oil in the absence of the ultrasound-assisted field (Control), respectively. The second and third weight losses correspond to the destruction of glycosidic bonds from sodium alginate [29].

In the case of sodium alginate, second degradation steps were observed in the temperature range of 220–340 °C, while for microcapsules with rapeseed oil (US and Control) this step ends at a higher temperature, 420 °C. The weight loss associated with the sodium alginate degradation seen around maxim temperature of 280 °C for alginate, 387 °C for microcapsules (Control) and 399 °C for microcapsules (US), respectively, was of 31.90%, 71.5% and 73.07%, respectively. The degradation step after 340 °C, associated also with the decomposition of the polymer network, had a mass loss of 7.95% at around 362 °C for sodium alginate, 20.17% at 456 °C for microcapsules (US) and 20.80% at 456 °C for microcapsules (Control) [30].

**Figure 5:**
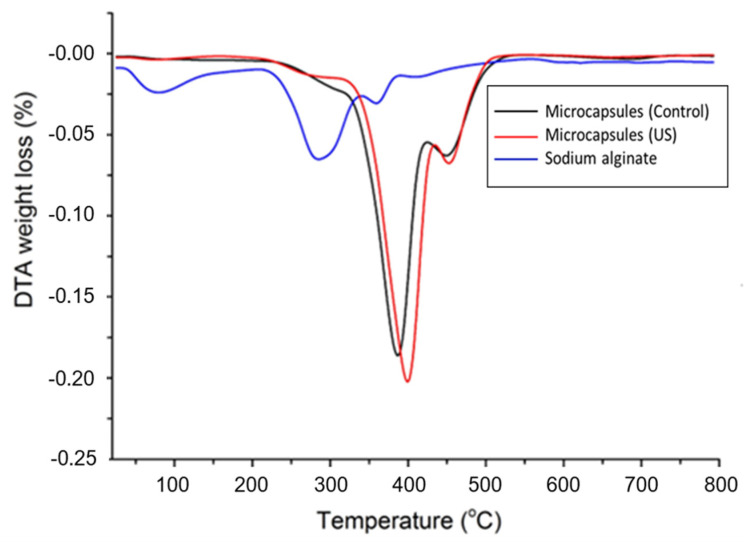
DTG thermograms for sodium alginate, microcapsules with rapeseed oil in the presence (US) and absence of ultrasound-assisted field (Control).

From the DTG curves (Figure 5), it can be observed that improved thermal stability of the microcapsules relative to the sodium alginate is caused by the cross-linking between sodium alginate and calcium ions, favored by the electrostatic interactions between the opposite charges of the individual compounds, therefore leading to the formation of structures with increased thermal characteristics [31]. It should be emphasized that the microcapsules with rapeseed oil in presence of an ultrasound-assisted field (US) had a higher thermal stability compared with classic method. This behavior was explained by Prasetyaningrum et al., who proposed a mechanism of the ultrasound-assisted encapsulation process [32]. The developed mechanism suggests that the US field, due to its specific alternative movement of the liquid phase, favors a better contact between the carboxyl group in alginate and the calcium ions (the “egg-box” model), thus increasing the probability of extra cross-linking, which, in turn, creates stability in the polysaccharide and in the microcapsules.

### 3.6. SEM Micrographs Analysis

**Figure 6:**
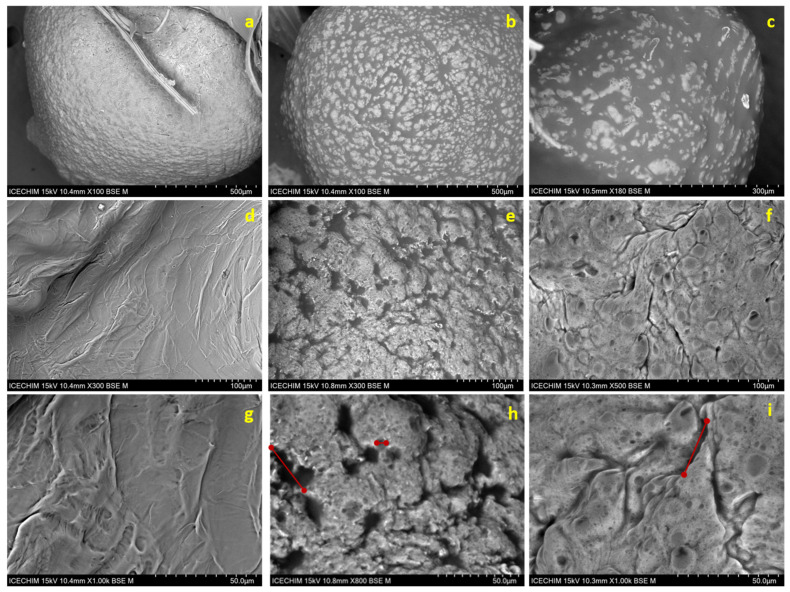
SEM morphology of sodium alginate (**a**,**d**,**g**), microcapsules with rapeseed oil in the presence of the US field (US) (**b**,**e**,**h**) and the absence of the US field (Control) (**c**,**f**,**i**).

In general, as can be seen in Figure 6, the dried beads regardless of the obtaining method showed an almost spherical shape and a rough surface, with the appearance of some wrinkles caused by the water loss during the drying process. However, it should be noted that, in the presence of the US field, the irregularities of the microcapsules’ surface increased, due to the specific movements of the continuous phase. Moreover, the SEM images show the absence of cracking in the particles produced which is important to ensure low gas permeability and enhanced protection of the encapsulated oil against oxidation [33].

This fact suggests that the addition of tween emulsifier stabilized the emulsion between the hydrophilic compounds (e.g., water, alginate) and oil, the hydrophobic compound [32]. Figure 6b,c shows that oil-loaded microcapsules obtained in the presence of the US field (US) and the absence of the US field (Control), compared to the empty one (Figure 6a), presented a crimpy surface with visible lumps. This observation agrees with the study developed by Soliman et al., who explained this phenomenon based on the deposition of oil droplets on the outer or inner surface of the external layer of alginate microbeads, where the presence of oil components on the alginate surfaces can lead to plasticization of their structure and the formation of these lumps during drying process [16]. By magnifying at a scale of 100 µm on the local area of the microcapsules, it could be seen that the empty microcapsule (Figure 6d), has a compact surface with no pores or cracks.

Regarding the microcapsules loaded with oil obtained in the US field (Figure 6e), although cavitation phenomena can cause cracks and pores on the surface of the sonicated microcapsules leading to the mechanical degradation of the layer wall material, the SEM images exhibited a good encapsulation structure with a slightly wrinkled surface with pores distribution length in the range 5–33 µm (Figure 6h) [32]. The microcapsules obtained without US treatment (Figure 6f) present a dense surface with distinct wrinkles and ridges. The length of the pores was between 3 and 30 µm (Figure 6i).

### 3.7. Microcapsule Morphology Analysis and Size Determination

The size of the microcapsules obtained under optimal conditions in the presence of the US field (US) and in the absence of the US field (Control) ranged between 0.74–0.81 mm and 1.04–1.16 mm, respectively (Table 4). For the best replicability of the results, all measurements were performed in duplicate.

Also, for the same experiments, the values regarding the sphericity factor were 0.0451 ± 0.01 and 0.0545 ± 0.02, respectively. From Table 5, it can be observed that the microcapsules obtained in the presence of the US field have a smaller size and sphericity factor. These microcapsules are more uniform in shape and size, having a good spherical shape. The results showed that the ratio of oil to sodium alginate and the higher concentration of calcium chloride solution had a greater influence on the shape of the microcapsules. Also, making microcapsules in the presence of a US field led to microcapsules of smaller and more uniform sizes.

In the studies carried out by Chan et al., the change in the shape of the microcapsule is better explained using the sphericity factor [20]. They consider that a droplet/microcapsule is considered spherical if the sphericity factor <0.05. This is because the extent of deformation cannot be clearly distinguished by human vision [33]. The microcapsule images obtained with the digital microscope are also consistent with the SEM images.

### 3.8. Encapsulation Efficiency

Microencapsulation of bioactive compounds is a process in which these compounds are enclosed in microscopic structures or capsules to protect them from external factors and to control their release in a specified manner. Therefore, it is essential to establish the capacity of the polymer matrix to retain bioactive compounds, as a quantifiable way to achieve the optimal dose of encapsulated bioactive compounds. The oil fraction on the wet surface of the microcapsules was determined, as well as the amount of oil encapsulated in the alginate matrix.

As observed by Bannikova et al. in the present study, the EE depends on the degree of crosslinking at the surface of the extruded emulsion droplet [15]. Excessive amounts of oil in an alginate solution prevent sufficient crosslinking once the emulsion droplet is immersed in a calcium chloride bath, leading to the formation of a highly porous wall.

The good results regarding the EE of the microcapsules (produced in the presence and absence of the US field) ranged between 76.56 ± 0.02% and 75.98 ± 0.02% (Table 6). The maximum efficiency (76.56 + 0.03%) was obtained for optimal conditions (US), results that are also in agreement with the those previously reported by Chan et al. [33]. The lowest encapsulation efficiency (43.05 + 0.02%) was found for the experiment with the parameters sodium alginate/rapeseed oil ratio of 5, calcium chloride concentration of 2.25% in a dripping time of 15 min in the presence of the US field (US). In the absence of the US field (Control) for the same conditions an encapsulation efficiency of 42.43 + 0.01% was obtained. The high ratio of sodium alginate to oil had a significant effect on the EE of the microcapsules. The low EE of microcapsules with a lower alginate/oil ratio can thus be attributed to an insufficient amount of coating material, which results in an inadequately strong structural matrix. Also, the thinner layer of wall material between the encapsulated oil and the short encapsulation time led to droplet destabilization during microcapsule formation [33]. This effect is amplified by longer dripping times, which favor bigger microcapsules, leading to a larger external surface of each drop, increasing the time of external layer formation.

### 3.9. Total Phenolic Content and Antioxidant Activity

The total content of phenols (such as flavonoids and phenolic acid) found in rapeseed oil contributes significantly to the antioxidant activity of the oil. Rapeseed oils containing a higher concentration of phenols have stronger antioxidant activity, which makes them beneficial for human health, helping to prevent the harmful effects of oxidative stress [13].

The total phenolic content was determined and then, the antioxidant activity of the samples was evaluated using the DPPH method. According to Table 7, a clear inverse correlation is observed; samples with a higher content of phenols in the case of microcapsules obtained in the presence of the ultrasound field (324.85 ± 0.01 mg GAE/g) have a higher antioxidant activity (lower IC50, 57.05 ± 0.02 mg/mL).

This confirms that phenols in microcapsules obtained in the US field (US) are more effective in neutralizing free radicals. US treatment could increase the extraction or availability of bioactive phenols, which explains better antioxidant activity. A possible explanation would be the effect of the interactions of free radical species, generated during cavitation (the main characteristic of the effects of the US field upon the carrier liquid phase), with the phenols from rapeseed oil. In the case of propolis extracts, the polyphenolic profile was affected by the US field, with consequences on the antioxidant and antimicrobial activity [34]. It could be possible that, during encapsulation, the US field propagating through microcapsules, which harden progressively, generates cavitation inside them, thus interfering with the polyphenolics due to the generated radicals, changing their profile, hence, the antioxidant activity [35]. However, this was not the goal of the present research.

A strong negative correlation was observed between total phenolic content (TPC) and IC50 values (Pearson’s r = −0.998), indicating that higher phenolic content corresponds to increased antioxidant activity. This suggests that phenolic compounds significantly contribute to the antioxidant potential of the samples.

If the IC50 value for microcapsules obtained in the US field (US) is lower than for those obtained in the absence of the ultrasound field (Control) (79.98 ± 0.04 mg/mL), it means that microencapsulation by ultrasound improved the efficiency of phenols, either by increasing the extraction or by structural modification that increased their antioxidant activity. This result would indicate that the use of a US field in obtaining microcapsules could be an effective method to improve the stability and antioxidant functionality of phenols in microencapsulated rapeseed oil.

## 4. Conclusions

In this paper, the microencapsulation process of bioactive compounds from rapeseed oil using sodium alginate in the presence and absence of an ultrasonic field is reported. A Box–Behnken experimental design is used to investigate the influence of process parameters on the microencapsulation process, which is then optimized, according to the response surface methodology, thus finding the operating conditions for which the yield is maximum.

The main difference between the two microencapsulation processes is the hydrodynamic state of the calcium chloride solution, contained in the beaker.

During the Control experiment, the solution contained in the beaker was under magnetic stirring (500 rpm), creating a velocity field which has the tendency of elongating the drops which enter in contact with this solution. Therefore, larger sphericity factors appeared.

During the experiments where the beaker is placed into the sonication bath, the US field passes through the glass, losing some intensity, since a part of the radiation is reflected, and forms complex standing waves in the calcium chloride solution, with a velocity field completely different from the Control experiment. Due to the interactions between the microcapsules, which harden progressively, and this complex velocity field, the outer surface becomes wrinkled (larger specific area), the particle tends to keep its spherical form (smaller sphericity factor), while the internal structure of the calcium alginate walls is affected, compared to Control. Optical microscopy, FTIR and SEM analysis confirmed that the rapeseed oil-containing microcapsules obtained in the presence of the ultrasonic field (US) were uniformly distributed in the wall material structure. Therefore, the effects of the ultrasonic field on the microencapsulation process, resulting from the present study, can be summarized as follows:

- Higher microencapsulation yields and efficiencies;
- Increased total phenolic content;
- Higher antioxidant capacity;
- Smaller sphericity factors and more uniformity in size distribution, providing better control over the release of active substances.

Under optimal operating parameters, the corresponding microencapsulation efficiency obtained was 90.25 ± 0.02% in the presence of the US field, and 87.11 ± 0.02 % in the absence of the ultrasound field (Control), respectively, results close to the predicted values given by the polynomial models.

This research showed the high potential of the microencapsulation of bioactive compounds process subjected to a US field.

Future investigations may focus on elucidating the effects of the radical species generated by cavitation (calcium chloride solution contains mainly water, which is prone to cavitation, while internal walls amplify it) interacting with the encapsulated oil, which could alter the polyphenolic species distribution, thus giving a solid explanation for the gain in total phenolic content and, consequently, in antioxidant activity.

## Figures and Tables

**Table 1:** Encapsulation parameters, microcapsules obtained and the yield for the microencapsulation process.

Mass Ratio, *w*/*w*	CaCl_2_ Concentration, %	Dripping Time, min	Flow Rate, mL/min	Microcapsules, g		Encapsulation Yield, %	
				US	Control	US	Control
5	1.5	22.5	0.89	15.21 ± 0.02	15.4 ± 0.02	76.05 ± 0.02	77.00 ± 0.02
5	3	22.5	0.89	16.19 ± 0.04	16.58 ± 0.02	80.95 ± 0.03	82.9 ± 0.02
10	1.5	22.5	0.89	15.43 ± 0.04	15.16 ± 0.02	77.15 ± 0.02	75.8 ± 0.02
10	3	22.5	0.89	16.08 ± 0.02	16.16 ± 0.04	80.4 ± 0.02	80.8 ± 0.02
5	2.25	15	1.33	16.41 ± 0.04	16.45 ± 0.03	82.05 ± 0.02	82.25 ± 0.02
5	2.25	30	0.67	16.99 ± 0.03	16.87 ± 0.04	84.95 ± 0.03	84.35 ± 0.03
10	2.25	15	1.33	16.16 ± 0.02	15.62 ± 0.03	80.8 ± 0.02	78.1 ± 0.02
10	2.25	30	0.67	17.38 ± 0.04	16.85 ± 0.04	86.9 ± 0.03	84.25 ± 0.03
7.5	1.5	15	1.33	16.37 ± 0.03	15.4 ± 0.02	81.85 ± 0.02	77.00 ± 0.02
7.5	1.5	30	0.67	17.23 ± 0.04	16.74 ± 0.04	86.15 ± 0.03	83.70 ± 0.02
7.5	3	15	1.33	16.95 ± 0.03	15.82 ± 0.02	84.75 ± 0.03	79.10 ± 0.02
7.5	3	30	0.67	17.87 ± 0.04	17.09 ± 0.04	89.35 ± 0.03	85.45 ± 0.03
7.5	2.25	22.5	0.89	17.83 ± 0.04	17.28 ± 0.04	89.15 ± 0.03	86.40 ± 0.03
7.5	2.25	22.5	0.89	17.84 ± 0.04	17.26 ± 0.04	89.10 ± 0.03	86.3 ± 0.03

**Table 2:** The optimal and experimental results.

Microencapsulation Process	Optimal Results					
	Estimated					Experimental
	R^2^	x_1_	x_2_	x_3_	Y	Y
US	0.996	8.07	2.57	28.1	90.67	90.25 ± 0.02
Control	0.962	8.01	2.54	25.7	87.51	87.11 ± 0.02

**Table 3:** Fatty acid composition of microencapsulated rapeseed oils.

Fatty Acids	Composition, %
Saturated fatty acids	
Palmitic (C16:0)	7.3 ± 0.02
Stearic (C18:0)	1.22 ± 0.0
Monounsaturated fatty acids	
Oleic (C18:1)	61.76 ± 0.09
Eicosanoic (C20:1)	3.44 ± 0.01
Erucic (C22:1)	1.56 ± 0.0
Polyunsaturated fatty acids	
Linoleic (C18:2)	15.22 ± 0.03
Linolenic (C18:3)	9.5 ± 0.02

**Table 4:** The assignment of peaks in the ATR-FTIR spectra.

Peak	Wavenumber, cm^−1^	Functional Group	Vibration Mode
1	2923	CH_2_^−^	stretching vibration
2	1744	C=O	stretching vibration
3	1461	–CH_2_	bending vibration
4	1160	C–O–C	symmetric stretching vibration
5	742	C=O	stretching vibration
6	1628	–COO–	stretching vibration
7	1451	–COO–	stretching vibration
8	1066	C–OH	stretching vibration
9	1635	–COO–	stretching vibration
10	2853	–CH–	stretching vibration

**Table 5:** Characteristics of rapeseed oil microcapsules for optimal obtaining parameters.

Microcapsules	Size, mm	Sphericity Factor (SF)	Microcapsules Image	Micrograph Image *
US	0.81 ± 0.01 ^b^	0.0451 ± 0.01 ^b^	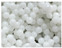	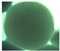
Control	1.16 ± 0.02 ^a^	0.0545 ± 0.02 ^a^	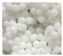	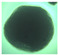

The value associated to b is statistically lower than the value associated to a. * The sizes of the microcapsules in the images appear the same but are of different sizes. For better resolution, the pictures were taken from different distances.

**Table 6:** Encapsulation efficiency values.

Mass Ratio, *w*/*w*	CaCl_2_ Concentration, %	Time, min	Encapsulation Efficiency (EE), %	
			US	Control
5	1.5	22.5	45.05 ± 0.02	44.43 ± 0.01
5	3	22.5	61.98 ± 0.01	62.75 ± 0.02
10	1.5	22.5	47.21 ± 0.02	44.56 ± 0.01
10	3	22.5	59.84 ± 0.01	60.66 ± 0.01
5	2.25	15	43.05 ± 0.02	42.43 ± 0.01
5	2.25	30	58.23 ± 0.01	57.95 ± 0.02
10	2.25	15	65.21 ± 0.02	51.45 ± 0.02
10	2.25	30	70.12 ± 0.01	69.37 ± 0.03
7.5	1.5	15	62.22 ± 0.01	55.08 ± 0.02
7.5	1.5	30	74.11 ± 0.01	72.65 ± 0.01
7.5	3	15	72.75 ± 0.02	69.76 ± 0.02
7.5	3	30	74.54 ± 0.01	73.45 ± 0.03
7.5	2.25	22.5	75.23 ± 0.01	72.35 ± 0.02
7.5	2.25	22.5	75.18 ± 0.01	72.98 ± 0.02
8.07	2.57	28.1	76.56 ± 0.02	-
8.01	2.54	25.7	-	75.98 ± 0.02

**Table 7:** Total phenolic content and antioxidant activity.

Microcapsules	Total Phenolic Content, mg GAE/g	IC50, mg/mL
US	324.85 ± 0.01 ^a^	57.05 ± 0.12 ^b^
Control	97.58 ± 0.02 ^b^	79.98 ± 0.07 ^a^

The value associated to b is statistically lower than the value associated to a.

## Data Availability

The original contributions presented in the study are included in the article; further inquiries can be directed to the corresponding authors.
